# Life History Orientation Predicts COVID-19 Precautions and Projected Behaviors

**DOI:** 10.3389/fpsyg.2020.01857

**Published:** 2020-07-24

**Authors:** Randy Corpuz, Sophia D’Alessandro, Janet Adeyemo, Nicole Jankowski, Karen Kandalaft

**Affiliations:** Department of Psychology, University of Massachusetts Boston, Boston, MA, United States

**Keywords:** COVID-19, coronavirus, life history theory, pandemic, conservatism

## Abstract

The ongoing Coronavirus disease (COVID-19) pandemic has had a devastating impact worldwide. It is unclear as to what one expects during the “post-peak” and “post-pandemic” periods in terms of: (1) continued adherence to precautionary measures (e.g., wearing a mask) and (2) behaviors during these periods pertaining to widespread (anticipated) medical solutions that can buffer subsequent waves (e.g., vaccination and donating plasma). In this study, we examine predictors of individual differences in attitudes and behaviors with regard to the COVID-19 pandemic and the months moving forward. Of the factors that contribute to how one might navigate the pandemic – a source of elevated environmental threat – life history orientation may play a crucial role. In this study, participants (*n* = 209) indicated their agreement with items on attitudes toward COVID-19 precautions and medical solutions that can buffer subsequent waves. In all models, we found significant positive relationships between one’s slow life history orientation and their self-reported adherence to precautions and endorsement of medical solutions. This effect was detectable even after controlling for factors related to political conservatism and personal experience with deleterious events as a result of the pandemic. Discussion includes reflection on the main finding, demographic variables, as well as the relationships uncovered among the modeled covariates (e.g., social conservatism, political conservatism).

## Introduction

The ongoing Coronavirus disease (COVID-19) pandemic has – in the United States alone – infected over 1.5 million people and has claimed the lives of more than 100,000 individuals (CDC as of June 1, 2020; cdc.gov/coronavirus/2019). For some, this information is sufficient to cause alarm and bring attention to things one can do to avoid infection and/or infecting others. Yet, there lacks uniform agreement as to what to expect during the “post-peak” and “post-pandemic” periods in terms of: (1) continued adherence to precautionary measures (e.g., wearing a mask) and (2) behaviors during these periods pertaining to widespread (anticipated) medical solutions that can buffer subsequent waves (e.g., vaccination and donating plasma). We explore these gaps in detail using data collected at the height of the pandemic.

### Life History Theory

When confronted with challenge or environmental uncertainty, one’s response is not arbitrary. At the core of life history theory (LHT)^1^ is the appreciation for the enduring influence of information in early development being utilized as a forecast in service of meeting the environmental demands of later development (Ellis and Bjorklund, 2012). Life history strategies exist along a “slow” to “fast” continuum – terms that indicate the relative tempo of one’s development and reproduction (Ellis and Bjorklund, 2012). Slow strategists are characterized by stable relationships (kin, romantic, social exchange partners) and a propensity for long term planning, risk averseness, and prosocial behavior (Del Giudice and Belsky, 2011). When the early environment presents itself as safe and stable, one can be assured that their life (and the lives of those around them) will extend well into the future. Fast life histories are marked by the opposite pattern. Faced with the risk of premature death and forced to navigate a social environment with exploitative agents, fast strategists accelerate development and develop an orientation toward succeeding in the here and now. These include risk taking, short term decision making, and decreased prosociality; these strategies are highly adaptive in environments where life is uncertain (Ellis et al., 2003; Simpson and Belsky, 2008; Del Giudice and Belsky, 2011).

In higher risk environments, one’s “fast” life history strategy will be comprised of being less averse to risk, more present (as opposed to future) oriented, and less affiliative (de Baca et al., 2016; Zhu et al., 2018; Lu and Chang, 2019). This self-centered, antagonistic social strategy helps the individual prepare for competing with others that may have divergent interests in their own immediate survival. Under risky and unpredictable environmental conditions, one must attend to immediate survival needs and discount future interactions and conspecific cooperation.

In lower risk environments, one can execute social strategies that are more mutualistic as one can rely on the convergent interests of those in a group (Figueredo and Jacobs, 2010; Chang and Lu, 2018; Lu and Chang, 2019). In environments that are more predictable, slow strategists can orient toward long term planning and more affiliative social behaviors (Ellis et al., 2009; Figueredo et al., 2018). One can afford to invest in a social orientation that includes coexistence and cooperation with others to maximize resource acquisition through collaboration (Chen and Chang, 2016; Zhu et al., 2018, Zhu and Chang, 2019).

### Predictions

There are differences in the levels of attitudinal endorsement for behaviors that can reduce the impact of COVID-19 (e.g., prolonged social distancing, masks in public) and those that can help buffer the impact on mortality (e.g., vaccines, plasma donation from recovered) in the long term. As detailed above, slow life history strategists demonstrate a social orientation toward longer term planning, more affiliative social behaviors, and being risk averse. *We hypothesize that those who demonstrate a slow life history strategy will have higher levels of attitudinal endorsement for behaviors that can reduce the impact of COVID-19.*

Similarly, we hypothesize that slow life history strategists would be more likely to endorse more affiliative behaviors such as donating plasma and vaccine administration. We include covariates where appropriate to isolate the strength and direction of this relationship. These include demographic variables (age, sex, religion, geographical location) as well as personal experience with pandemic-related events and political conservatism – factors that play a role in attitudes and behavior during pandemics (e.g., Navarro et al., 2006; Mesch and Schwirian, 2015; Barrios and Hochberg, 2020).

## Methods

### Overview and Study Design

This study was explicitly designed in response to the COVID-19 pandemic. Participants contributed data between May 9, 2020 and May 19, 2020^2^. All materials and procedures were reviewed and approved by the University’s Institutional Review Board (IRB).

This study utilizes a convenience sample from Amazon’s Mechanical Turk (MTurk), an online survey tool. MTurk is particularly well-equipped to collect data from participants remotely. MTurk participants demonstrate psychometric equivalence to other data collection methods (Paolacci et al., 2010; Arditte et al., 2016; Hauser and Schwarz, 2016; Kees et al., 2017; McCredie and Morey, 2019).

Utilizing G^∗^Power (Erdfelder et al., 1996), we determined an optimal sample size for this fixed model with an anticipated “small effect” and up to six predictors (power = 0.95) would be at least *n* = 146. The outcome measures utilized in this study have not been deployed together; we were conservative with our prediction of effect size when calculating *a priori* sample size.

Participants were presented with a consent form and granted consent prior to seeing any items from the survey. At the conclusion of one’s participation, they were compensated $11.50 for completion of the study materials, which included additional measures not examined in the present study (573 total items; mean response time = 77 min).

### Participants

Participants (*n* = 209) contributed data between May 9, 2020 and May 19, 2020. The sample was disproportionally male (55.1%). Participants ranged from 19 to 60 years of age, with a mean age of *M* = 33.4 years (SD = 11.4). The majority of participants identified as European American (72.5%), followed by Asian American (9.7%), African American (7.3%), Hispanic or Latino/a (6.1%), Native American (1.5%), and Other (0.9%). In terms of religion, this sample lacked the heterogeneity (i.e., Agnostic/none = 53% and Christian, 40%; maximum of *n* = 2 in remaining groups) to form any more than two groups and, as a result, religion will be treated as a binary variable. The median income of this sample was reported as $45,000–$60,000 annually. In terms of education, 62.2% of this sample held at least a bachelor’s degree. Based on census demarcations (see “Demographic Covariates” section), the geographical distribution of participants in this study were: Northeast (*n* = 54), South (*n* = 46), Midwest (*n* = 41), Pacific (*n* = 26), and Mountain (*n* = 6).

### Materials

#### Life History Strategy

The Life History Battery Short Form (LH-SF; Figueredo et al., 2015) assesses several domains of social and sexual behavior that reveal an individual’s “K-factor” – the degree to which one adopts a fast versus slow life history strategy. The LH-SF is a psychometric measure of life history orientation. Items ask about cognitive and behavioral indicators of one’s life history orientation. For example: “When faced with a bad situation, I do what I can to change it for the better” and “While you were growing up, how much love and affection did your biological father provide.” Participants respond to each question on a Likert scale ranging from strongly disagree (−3) to strongly agree (+3). The measure demonstrates convergent validity (Olderbak et al., 2014) and Cronbach’s alpha is consistently adequate in the literature (α > 0.70) (see Olderbak et al., 2014).

The LH-SF produces a total (summed) value across all items. In the current sample, reliability was adequately high as well (α = 0.91 across 42 items). Life history orientation was normally distributed (skewness = 0.03, SE = 0.17; kurtosis = −0.47, SE = 0.34). This predictor variable (*M* = 50.94, SD = 30.23) was treated as a continuous variable in all models. Higher values on this measure indicate a *slower* life history strategy.

#### COVID-19 Deleterious Events

The Epidemic-Pandemic Impacts Inventory (EPII; Grasso et al., 2013) was developed to learn more about how the pandemic has changed people’s lives. For each statement, participants indicate whether the pandemic has impacted the self or others in the household (or both) for each item. The EPII asks participants to self-report on the occurrence of stressors in various domains of personal and family life: *“We would like to learn how the coronavirus disease pandemic has changed people’s lives. For each statement below, please indicate whether the pandemic has impacted you or your family in the way described.* The full EPII is 107 items (α = 0.82 in current sample) distributed through 11 subscales. For the current study, we excluded the subscale “positive events” as our interests were on the influence of experiencing deleterious events. The full EPII can be found online: health.uconn.edu/psychiatry/child-and-adolescent-psychiatry-outpatient-clinic.

There are no psychometric properties yet available for the EPII and optimal scoring procedures are not yet determined. As a covariate in our model, we were only interested in the total cumulative exposure to deleterious events. The total number of deleterious events was normally distributed (skewness = 0.51, SE = 0.17; kurtosis = 0.66, SE = 0.34). This predictor variable (*M* = 19.39, SD = 7.86) was treated as a continuous variable in all models.

#### Political Conservatism

The 12-item social and economic conservatism scale (SECS; Everett, 2013) is a measure of political conservatism consisting of two subscales: social conservatism (5 items) and political conservatism (7 items). Higher scores indicate higher levels of conservatism on both scales. The SECS is presented to participants as a continuous scale slider that ranges from 0 (feeling extremely negative toward an issue) to 100 (feeling extremely positive toward an issue). Sample items for social conservatism (α = 0.86) include “Abortion” and “Patriotism” and sample items for the economic conservatism subscale (α = 79) include “Limited Government” and “Welfare Benefits.” The full SECS is available online: PLOS One doi: 10.1371/journal.pone.0082131

In this sample, the social (*M* = 261.15, SD = 180.57) and political (*M* = 167.81, SD = 112.13) scores were highly correlated (*r* = 0.64, *p* < 0.001). Both were normally distributed: social conservatism (skewness = 0.09, SE = 0.17; kurtosis = −0.96, SE = 0.34) and political conservatism (skewness = −0.02, SE = 0.17; kurtosis = −0.51, SE = 0.34). Higher scores indicate higher levels of conservatism on both scales. Both scales were modeled individually as exogenous predictor variables to maintain a conceptual distinction.

#### Demographic Covariates

There were four specific demographic variables that we anticipated having a relationship with our outcome variables: age, sex, religion, and geographic location (see section “Participants”).

Specific to geographic location, participants were given the option of reporting their zip code on MTurk; 82.7% of participants (*n* = 173) provided this information. These zip codes were broken up into geographical regions based on Census demarcation boundaries: Northeast (*n* = 54), South (*n* = 46), Midwest (*n* = 41), Pacific (*n* = 26), and Mountain (*n* = 6). We also used this information to find the “% rurality” compiled per county by the most recent census (2010 Census Rural County Lookup)^3^. This produces a continuous variable (0–100) that indicates how “urban” or “rural” a Census-delineated county was as of the last census (higher scores indicating higher rurality). The majority of this sample resided in counties characterized by low rurality (*M* = 14.31, SD = 18.19). There were no differences on study variables between those who provided zip code information and those who did not (*p*s > 0.34).

In an attempt to build a parsimonious model (increased df and less model parameters), we explored the bivariate relationship between each of the four demographic variables with the outcome variable in all models. While the decision to exclude variables in models need not only rely on statistical significance, we viewed this step as necessary to eliminate “impotent controls” prior to building a final model (see Becker, 2005) and to limit the number of estimated parameters whenever possible (i.e., Jackson, 2003).

#### Attitudes Toward COVID-19 Precautions

Participants were given a six-point Likert scale anchored with “strongly disagree to strongly agree.” Three items were presented to all participants in random order: “How much do you agree with the following statements: (1) Wearing masks in public spaces is necessary; (2) People should continue to stay-at-home (quarantine) even if COVID-19 cases start to fall; (3) The news and threat about COVID-19 is “overblown.” A latent outcome variable (named Attitude toward Precautions) was created using all three items on our custom measure. Item loadings ranged from.78 to.86 (all unstandardized estimates *p* < 0.001) which is sufficiently high enough to retain all items as indicators (Hair et al., 1998).

#### Pandemic Recovery Behavior

Participants were asked the single item “How willing would you be if asked to donate plasma (blood) to helping those with COVID-19?” They responded using an 11-pt scale (0–10) with higher scores indicating more willingness to donate. As a separate item, participants were asked: “How much do you agree with the statement: A vaccine for COVID-19 should be mandatory.” Both variables are maintained as continuous outcome variables through all models.

## Results

### Preliminary Analyses

**FIGURE 1:**
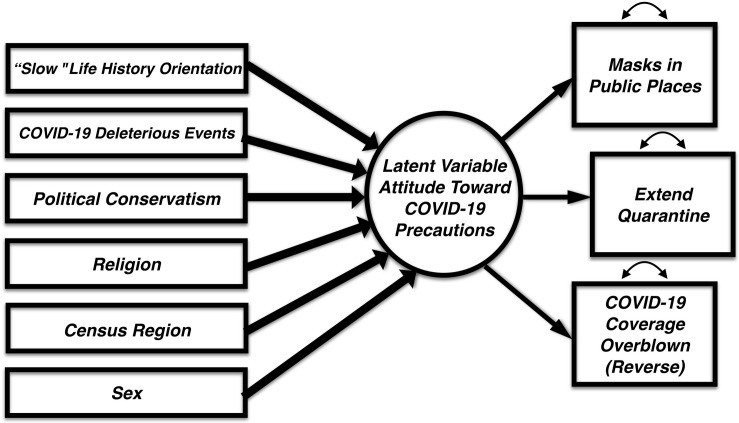
Proposed hybrid structural equation model including all model variables.

All analyses were run using SPSS (v. 22) and AMOS (v. 22). Prior to all analyses, variables were examined for normality (skewness, kurtosis, outlier identification). Overall, missingness was minimal with the exception of one item (zip code; see section “Methods”) which was elective for participants. We fitted all models using full information maximum likelihood (FIML) estimator using AMOS (v.22). Prior to model building, demographic items (sex, age, region, religion) were explored to identify whether it was appropriate (on statistical grounds) to maintain each in the model building process for each outcome variable examined. A zero order correlation table of all predictor variables is provided on Supplementary Table 1. The proposed a prior model can be found in Figure 1.

### Outcome: Predicting COVID-19 Attitudes Toward Precautions

#### Demographic Covariates

To examine demographic variables for inclusion in models predicting “Attitudes toward precautions,” we ran a series of mean comparisons. Sex (*p* = 0.11), religion (*p* = 0.34), age (*p* = 0.30), and geographical region (*p* = 0.12) were not related to the latent outcome variable. This was also the case when conceptualizing location as the “percentage of rurality” (*p* = 0.75). As a result, we excluded these variables to maintain model parsimony and sufficient degrees of freedom (Achen, 2005).

### Model Building and Testing

**FIGURE 2:**
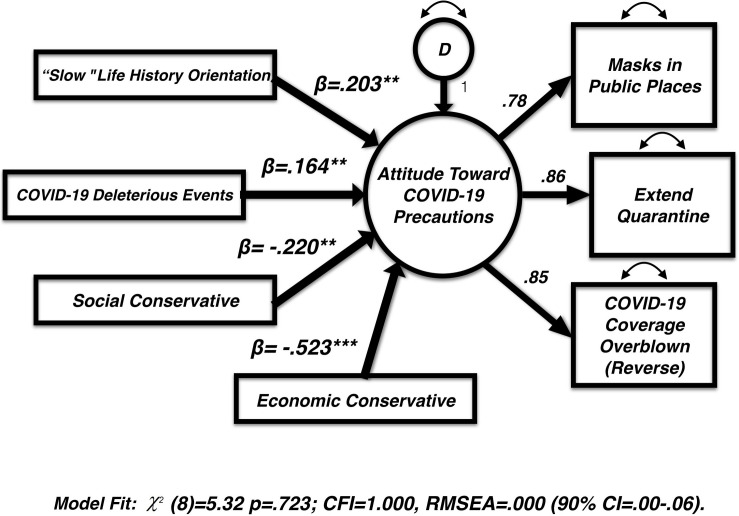
Final hybrid structural equation model including only variables retained. All endogenous (predictor) variables are modeled to covary with one another. Full covariate parameters are found on Table 1.

The resultant model (Figure 2) – whereby the latent variable “attitudes toward precautions” is predicted by life history orientation – was tested with the following covariates: social conservatism, economic conservatism, and COVID-19 deleterious events. The tested model fits the data well [(χ^2^(8) = 5.32, *p* = 0.723); CFI = 1.00, RMSEA = 0.000 (90% CI = 0.000–0.060)]. Life history orientation was able to predict a significant amount of variance in our latent variable “Attitudes Toward Precautions” (β = 0.20, *p* < 0.01). Those exhibiting a slow life history orientation were more likely to endorse precautions. This relationship was detectable even after controlling for social and economic conservatism and one’s self-reported occurrences of deleterious events resulting from COVID-19. All parameter estimates appear in Table 1.

**TABLE 1:** Parameter estimates for modeled predictors of the latent variable Attitudes Toward COVID-19 precautions.

Parameter	Unstd.	SE	Critical ratio	*p*	Std.
**Predictor effect**					
Slow life history	0.010	0.003	3.136	0.002	0.203
Deleterious events	0.030	0.011	2.791	0.005	0.164
Social conservative (SC)	–0.002	0.001	–2.695	0.007	–0.220
Economic conservative (EC)	–0.007	0.001	–6.591	***	–0.523
**Modeled covariance**					
Slow life history ↔ SC	2222.865	407.508	5.455	***	0.409
Slow Life History ↔ EC	481.999	236.636	2.037	0.042	0.143
Slow life history ↔ events	54.86	16.858	3.254	0.001	0.232
SC ↔ EC	12902.66	1659.076	7.777	***	0.640
SC ↔ events	128.392	98.355	1.305	0.192	0.091
EC ↔ events	–36.456	60.88	–0.599	0.549	–0.042

This model fit the data well: [(χ^2^(8) = 5.32, p = 0.723); CFI = 1.000, RMSEA = 0.000 (90% CI = 0.00–0.06)]. ***p < 0.001. “SC” = Social Conservatism; “EC” Economic Conservatism; “Events” – deleterious events as a result of COVID-19.

There are other significant relationships in this model that warrant analysis and discussion to a broader audience. In terms of the number of deleterious events, those self-reporting more events were more likely to endorse COVID-19 precautions (β = 0.16, *p* < 0.01). There were strong, negative effects for economic (β = −0.52, *p* < 0.001) and social conservatism (β = −0.22, *p* < 0.001) on the latent variable “Attitudes toward precautions.” In both cases, participants who reported being more conservative were also *less* likely to endorse precautions surrounding COVID-19. Path estimates and covariance values for modeled variables in Table 1.

### Outcome: Predicting COVID-19 Pandemic Recovery Behavior

Similar testing procedures were used in developing a model that could examine predictors of pandemic recovery behaviors (i.e., plasma donation and vaccine requirements). Each item is tested as an outcome variable separately. The analysis plan will remain consistent: (1) evaluate demographic variables for inclusion in final model; (2) run model to obtain parameter estimates. The analysis for both outcome variables will be equivalent to that of multiple regression with the added benefit of including FIML estimates for missing values (AMOS v22).

#### Donating Plasma

Sex (*p* = 0.91), religion (*p* = 0.78), location (*p*s > 0.38), and age (*p* = 0.45) were not related to this “donating plasma” variable and were excluded from models to maintain model parsimony (Achen, 2005). The resultant model – whereby life history orientation predicts one’s likelihood to donate plasma – included only the following covariates: social conservatism, economic conservatism, and COVID-19 deleterious events.

In this model, life history orientation was able to predict variance in one’s likelihood to donate plasma (β = 0.27, *p* < 0.001). This relationship was detected after controlling for social and economic conservatism and one’s self-reported occurrences of deleterious events resulting from COVID-19. Those exhibiting a slow life history orientation were more likely to report being willing to donate plasma.

In terms of the predictors that accompany life history orientation in this model: those self-reporting more events were more likely to indicate that they would donate plasma (β = 0.19, *p* < 0.01). Specific to donating plasma, economic conservatism had no relationship to this outcome variable (β = −0.02, *p* = 0.80). Social conservatism was however related to one’s reported likelihood of donating plasma (β = −0.22, *p* = 0.02) – those higher in social conservatism indicated less interest in donating plasma. Parameter estimates appear on Table 2.

**TABLE 2:** Parameter estimates for modeled predictors of (1) Donating Plasma variable and (2) Vaccine variable.

Parameter	Unstd.	SE	Critical ratio	*p*	Std.
**1. Predictor effect donate plasma**					
Slow life history	0.030	0.008	3.629	***	0.266
Deleterious events	0.080	0.029	2.805	0.005	0.164
Social conservative (SC)	–0.004	0.002	–2.404	0.016	–0.223
Economic conservative (EC)	–0.001	0.003	–0.260	0.765	–0.022
**2. Predictor effect mandatory vaccine**					
Slow life history	0.012	0.001	–2.067	0.039	0.159
Deleterious events	0.027	0.018	1.502	0.133	0.094
Social conservative (SC)	–0.002	0.001	–2.067	0.039	–0.179
Economic conservative (EC)	–0.007	0.002	–4.463	***	–0.357
**Modeled covariance**					
Slow life history ↔ SC	2222.865	407.508	5.455	***	0.409
Slow life history ↔ EC	481.999	236.636	2.037	0.042	0.143
Slow life history ↔ events	54.86	16.858	3.254	0.001	0.232
SC ↔ EC	12902.66	1659.076	7.777	***	0.640
SC ↔ events	128.392	98.355	1.305	0.192	0.091
EC ↔ events	–36.456	60.88	–0.599	0.549	–0.042

“SC” = Social Conservatism; “EC” Economic Conservatism; “Events”-deleterious events as a result of COVID-19; ***p < 0.001. Modeled covariance parameter estimates are identical to latent variable Attitudes Toward COVID-19 precautions.

#### COVID-19 Vaccination

Sex (*p* = 0.54), religion (*p* = 0.32), location (*p*s > 0.41), and age (*p* = 0.52) were not related to this “mandatory vaccination endorsement” variable and were excluded from models to maintain model parsimony (Achen, 2005). As with the model above, all remaining predictors were modeled to covary with one another.

In this model, life history orientation was able to predict variance in one’s level of endorsement for the statement “vaccination for COVID-19 should be mandatory” (β = 0.16, *p* = 0.04). This relationship was detected after controlling for social and economic conservatism and one’s self-reported occurrences of deleterious events resulting from COVID-19. Those exhibiting a slow life history orientation were more likely to endorse mandatory vaccination for COVID-19.

In terms of the predictors that accompany life history orientation in this model: those self-reporting more deleterious was not related to one’s indication that a vaccination for COVID-19 should be mandatory (β = 0.09, *p* = 0.13). Specific to the statement “vaccination for COVID-19 should be mandatory,” both economic conservatism (β = −0.36, *p* < 0.001) and social conservatism (β = −0.18, *p* = 0.04) were negatively correlated with one’s level of endorsement for this outcome. Those self-reporting as higher in conservatism were less likely to endorse the statement “A vaccine for COVID-19 should be mandatory.” Parameter estimates appear on Table 2.

## Discussion

In this study, we present evidence that one’s life history orientation contributes to which attitudes and behaviors one endorses in the face of an ongoing global pandemic. We utilized three separate outcome variables (attitude toward COVID-19 precautions, willingness to donate plasma, and opinions on COVID-19 vaccination). In each of these models, slow life history strategists adopted a more precautious and prosocial stance (i.e., long term planning). We did not find evidence that sex, age, religiosity, or geographical region (“rurality”) had any significant relationship with the outcome variables in this study.

Our results are consistent with extant literature on slow life history strategies and adherence to (and encouragement of) social and moral rules (Gladden et al., 2009). Social and moral rules increase social stability and help maintain a risk-averse environment for one’s community. Individuals routinely deploy moral emotions (e.g., anger, disgust) aimed at ensuring the upholding of rules and social contracts (Haidt and Bjorklund, 2008). Interestingly, general disgust sensitivity is thought to have evolved to motivate the avoidance of dangerous pathogens and later coopted to function in a similar manner within the social domain (Navarrete and Fessler, 2006); one can exclude or punish a rule breaker (e.g., refusing to wear a mask) in order to facilitate in-group cohesiveness and to motivate pathogen avoidance. In the context of the current study, slow strategists strongly endorsed more precautious and prosocial behaviors. While we did not measure things like (for example) “disgust toward those not wearing a mask,” we expect that, specific to slow life history individuals, one’s endorsement of a behavior would be tied to their enforcement of the behavior.

In exploring the contributions of life history orientation, we uncovered relationships that may be of broad interest. In general, political conservatism (social and economic) demonstrated considerable influence on precautionary attitudes and vaccine endorsement; those high in conservatism were lower on endorsement of precautions and vaccines. With regard to plasma donation, economic conservatism (but not social) demonstrated a significant relationship – higher economic conservatism was associated with a lower likelihood of plasma donation. Recent COVID-19 work found that conservatives discount the mainstream media and downplay reports of the severity of the pandemic (Rothgerber et al., 2020). Our work aligns with this research. The decreased levels of endorsement for precautionary measures among conservatives may be a consequence of underestimating risk due to discounting media reports on COVID-19.

One may have predicted that political conservatism to be *positively* correlated with one’s level of endorsement of precautions. There is a sizable literature detailing the relationship between conservatism and disgust sensitivity and fear of contamination (see Terrizzi et al., 2013 for a meta-analysis). Pathogen prevalence is positively correlated with authoritarianism (Murray et al., 2013) and conformity (Murray et al., 2011) while negatively correlated with democratic ideals (Thornhill et al., 2009) and openness to experience (Schaller and Murray, 2008). Those high in conservatism should, according to this literature, demonstrate *increased* conformity to precautions. This would also be the same prediction from other research on conservatism that has found a positive relationship with adherence to social norms (e.g., Altemeyer, 1988), avoiding behaviors contrary to a group’s best interest (Triandis, 1994), and evidence that socially conservative value systems are partly characterized by submission to authority (see Ludeke et al., 2013).

It is not clear from our data precisely why political conservatism is negatively associated with all three outcome variables in context of extant work. There is some evidence that those who support politically conservative leaders and policies are more likely to believe that the “free market” system is most efficient and to treat this as an ideology of sort (Monteith et al., 2016; Day and Fiske, 2017). A perception of “imposing” on free market forces may be driving down endorsement of precautions, vaccine mandate, and plasma donation. It may also be the case that novel precautions such as wearing a mask or social distancing have yet to reach a critical mass (in the minds of those high in conservatism) as behaviors that are to be conformed to. Even when precautions are socially and/or legally enforced, adherence to these precautions will still rely on cognitive machinery that must identify a behavior as widespread enough where executing that behavior can accurately be tagged as “conforming.” Part of the slow strategy itself might be to hold a particularly high threshold for when once novel social behaviors (e.g., wearing a mask) become the norm. While speculative, it may be the case that the COVID-19 pandemic (and all that it brings) offers nuance to the study of conservatism unseen prior. More complex models that include some of these additional variables are needed to address these questions on political conservatism further.

### Limitations

Our sample was recruited from the online survey tool MTurk. This sample possessed features that went well beyond what one might find with a standard convenience sample of undergraduates: e.g., age, employment, detrimental pandemic events in a multitude of domains (e.g., work, paying mortgage, quarantine with children, geographical range). MTurk does have limitations however (potential misrepresentation-MacInnis et al., 2020). It is important to note that MTurk participants are typically comparable or better to other data sources (Hauser and Schwarz, 2016; Kees et al., 2017; McCredie and Morey, 2019). Nonetheless, our results may not generalize to the broader population as we did lack representative levels of diversity in terms of race and education. Future research will have to consider MTurk’s limitations and benefits.

While the sample size (*n* = 209) was more than adequate to test our *a priori* models, more complex models (e.g., mediation, moderation) with many more parameters to estimate would require a much larger sample size than used here. There is potential to convert some of our *a priori* models into *post hoc* mediational models in this dataset. Parameter estimates however would be almost meaningless without an adequate sample size to test those models. This approach is also an effort to avoid what Achen (2005) has called the “kitchen sink” approach to structural equation modeling. Moving and plugging in/out variables, drawing and deleting paths, or finding unjustified (on theoretical grounds) ways to improve model fit are structural equation model strategies that we avoid in this initial attempt.

There is ongoing debate about exactly what is being measured when deploying a measure of life history, specifically with humans (e.g., Stearns and Rodrigues, in press). The life history measure we used here is a psychometric measure of life history orientation that aligns with extant theory and is used widely across research disciplines (Olderbak et al., 2014). Future research should continue to explore the psychometric properties of the life history measure used here. In parallel, debate should proceed in identifying (precisely) which components of theory may apply to humans vs. non-humans.

### Future Directions

The COVID-19 pandemic has been accompanied by unprecedented appeals to the greater public for groupwide adherence to precautionary measures and widespread discussion of community-health based medical interventions to “flatten the curve.” In this study, we identified the role that life history orientation may play in individual differences related to important decisions around COVID-19 going forward.

These decisions are of great consequence. For example, Hou et al. (2020) found that if the timing of when to declare a quarantine were delayed by even 1.5–2 days, community spread becomes exacerbated. In this context, understanding the predictors of high or low levels of compliance to pandemic precautions or prosocial behaviors is critical. In a study on pandemic influenza, Ekberg et al. (2009) found that a major contributor to reducing disease transmission was the degree to which individuals voluntarily exhibited precautionary behaviors. As some locations re-open across the United States and complacency to precautions increases, the voluntary use of (for example) a mask may become a key contributor to stemming a second wave of the disease.

Pathogens expose vulnerabilities in immunocompetence; pandemics lay bare the workings of cognitive adaptations geared toward negotiating the social environment. Understanding the exact contributions to these types of decisions should pay large dividends on a global scale.

## Data Availability Statement

The raw data supporting the conclusions of this article will be made available by the authors, upon reasonable request.

## Ethics Statement

The studies involving human participants were reviewed and approved by the Institutional Review Board of the University of Massachusetts Boston. The patients/participants provided their written informed consent to participate in this study.

## Author Contributions

RC was the Principle Investigator of this study, supervised all activity, wrote the Institutional Review Board (IRB) proposal, evaluated progress on literature review and survey measures, monitored data collection, ran statistical analyses, and drafted this current publication. SD’A, JA, NJ, and KK contributed to hypotheses, to finding applicable measures for this study, to coding the various paper measures for Qualtrics formatting, and to reading over the final publication. SD looked over the IRB proposal, oversaw and contributed to the literature review (specifically compensation and remote data collection method), implemented coding and edits to the Qualtrics survey measures for accuracy and pacing, collected Census data to support analyses, proofread this current publication and wrote this statement. JA, NJ, and KK conducted a comprehensive literature review (for remote recruitment, mechanisms of COVID-19 and prior pandemics, and relation between anxiety and pandemics, respectively), and personally went through the Qualtrics survey multiple times to report on fixable errors. KK and JA also created and organized the Facebook advertisement for the study. All authors contributed to the article and approved the submitted version.

## Conflict of Interest

The authors declare that the research was conducted in the absence of any commercial or financial relationships that could be construed as a potential conflict of interest.

## Footnotes

1 As it is utilized contemporarily in research on, for example, human psychology and psychobiology.

2 World Health Organization declared COVID-19 a pandemic on March 11 (World Health Organization COVID-19 Landing, 2020) and the United States declared a state of national emergency on March 13.

3 The Census Bureau delineates urban areas after each decennial census. See https://www.census.gov/programs-surveys/geography/guidance/geo-areas/urban-rural.html
