# Supplementary material for: Life History Orientation Predicts COVID-19 Precautions and Projected Behaviors
Source: Front Psychol. 2020 Jul 24;11:1857. doi: 10.3389/fpsyg.2020.01857 (PMC7393224; doi:10.3389/fpsyg.2020.01857)
Supplement: Supplementary file 1 [file Table_1.docx]

| **Predictor** | **1** | **2** | **3** | **4** | **5** | **6** | **7** |
| --- | --- | --- | --- | --- | --- | --- | --- |
| **(1) Slow Life History** |  |  |  |  |  |  |  |
| **(2) COVID Deleterious Events** | 0.23** |  |  |  |  |  |  |
| **(3) Social Conservatism** | 0.41** | 0.09 |  |  |  |  |  |
| **(4) Economic Conservatism** | 0.14** | -0.04 | 0.64** |  |  |  |  |
| **(5) Rurality Index** | -0.07 | -0.03 | 0.09 | 0.12 |  |  |  |
| **(6) Sex** | 0.06 | 0.11 | 0.06 | -0.12 | 0.09 |  |  |
| **(7) Religion** | 0.46** | 0.24** | 0.53** | 0.17* | 0.06 | 0.13 |  |
| **(8) Age** | 0.05 | -0.05 | 0.06 | -0.07 | 0.18 | 0.15 | 0.25** |

***p*<.01, **p<*.05

**Supplementary Table 1.** Zero order correlations of model predictors including demographic variables that were evaluated for inclusion as covariates in all models.
